# Antibody Therapies for Progressive Multiple Sclerosis and for Promoting Repair

**DOI:** 10.1007/s13311-022-01214-x

**Published:** 2022-03-14

**Authors:** Joachim Havla, Reinhard Hohlfeld

**Affiliations:** 1 Institute of Clinical Neuroimmunology, University Hospital, LMU Munich, Munich, Germany; 2 Biomedical Center (BMC), Faculty of Medicine, LMU Munich, Martinsried, Germany; 3 Data Integration for Future Medicine (DIFUTURE) Consortium, LMU Munich, Munich, Germany; 4 Munich Cluster of Systems Neurology (SyNergy), Munich, Germany

**Keywords:** Progressive multiple sclerosis, Antibody therapy, CD20, PMS, Ocrelizumab, Rituximab

## Abstract

Progressive multiple sclerosis (PMS) is clinically distinct from relapsing–remitting MS (RRMS). In PMS, clinical disability progression occurs independently of relapse activity. Furthermore, there is increasing evidence that the pathological mechanisms of PMS and RRMS are different. Current therapeutic options for the treatment of PMS remain inadequate, although ocrelizumab, a B-cell-depleting antibody, is now available as the first approved therapeutic option for primary progressive MS. Recent advances in understanding the pathophysiology of PMS provide hope for new innovative therapeutic options: these include antibody therapies with anti-inflammatory, neuroprotective, and/or remyelination-fostering effects. In this review, we summarize the relevant trial data relating to antibody therapy and consider future antibody options for treating PMS.

**Supplementary Information:**

The online version contains supplementary material available at 10.1007/s13311-022-01214-x.

## Introduction

Multiple sclerosis (MS) is the most frequent chronic inflammatory demyelinating disease of the central nervous system (CNS). Progressive multiple sclerosis (PMS) is characterized by a relentless increase of disability that is not associated with clinical relapses as they occur in the relapsing remitting form of the disease (RRMS). There is increasing evidence that the pathological mechanisms of PMS and RRMS are different. Whereas relapses are thought to be caused by acute focal inflammation, relapse-independent progression is the clinical consequence of more diffuse inflammatory and neurodegenerative processes [1, 2]. This is supported by MRI evidence of a decrease in the number of new lesions and increasing atrophy in patients with PMS (PwPMS), as well as by clinical evidence of an increase in disability without focal inflammation [3]. According to the modified Lublin criteria [3], the previously held distinction between primary progressive and secondary progressive MS is no longer necessary; both forms are included in the category of progressive MS (PMS). This is supported by evidence that primary and secondary progressive MS lack distinguishing histopathological features [4].

Whereas the inflammation dominating the early phase of RRMS is probably still driven by peripheral immune processes, it is thought that in PMS there is a “compartmentalization” of inflammation within the CNS compartment with a predominance of chronic-active, spreading and inactive lesions [1]. In addition, cortical and gray matter lesions, as well as meningeal lymphoid B-cell aggregates may act as drivers of disability progression [4]. Furthermore, increasing evidence points to microglial activation as a driver of progression. This seems to involve increased production of reactive oxygen species or nitric oxides [2]. This oxidative stress affects axonal mitochondria, leading to alterations of mitochondrial DNA and neuroaxonal energy deficiency [2]. Oxidative stress also affects the remyelination capacity of oligodendrocytes [5].

## General Aspects of Antibody-Based Therapies in PMS

### Anti-inflammatory Versus Neuroprotective Therapeutic Strategy

In view of the current concepts of the pathogenesis of PMS (see the “Introduction” section), it makes sense to consider two diferent treatment approaches: one aiming to curb inflammation and a second aiming to foster neuroprotection, remyelination, and repair. Because there is evidence that inflammation and neurodegeneration occur concomitantly from onset, theoretically one would like to combine these strategies from the beginning. Currently there is a lack of effective, sensu-stricto neuroprotective therapies. In this regard, it is worthwhile to distinguish between directly neuroprotective therapies and indirectly neuroprotective effects of anti-inflammatory interventions. By reducing pathogenic inflammation, anti-inflammatory agents help to preserve myelin and axons, thereby indirectly exerting protection.

### Time Window of Opportunity

Our understanding of the pathogenesis of PMS would seem to support a time-window-adjusted treatment strategy [6]. Indeed, phase III studies have consistently demonstrated that the time window of opportunity for anti-inflammatory medications appears to be the early phase of PMS. In addition, it was shown that younger age, shorter duration of PMS, and more pronounced clinical and MRI activity at baseline were more likely to be associated with a positive outcome [7, 8]. Therefore, anti-inflammatory therapies should be used at a disease stage dominated by an inflammatory pathomechanism. Consistent with these considerations, the currently available therapies seem to be effective mainly in the active phases of PMS, defined by superimposed relapses and focal MRI activity [7, 8]. Ideally and theoretically, anti-inflammatory therapies should act not only in the periphery but also directly in the central nervous system (CNS).

In contrast, one might predict that neuroprotective, reparative, and remyelinating therapies, should they become available in the future, may possess a larger time window of opportunity. One important potential therapeutic goal would be the activation and differentiation of oligodendrocyte progenitor cells (OPCs), increasing their potential to differentiate into myelinating oligodendrocytes [9]. Additional therapeutic aims include neuroprotection, e.g., by inhibiting apoptosis and oxidative stress, and protection of mitochondria [6].

### Risk–Benefit Ratio

The use of monoclonal antibodies in the treatment of MS has made it possible to target selected molecules thought to play a key role in the pathogenesis. In RRMS antibody, therapy is associated with a high level of efficacy regarding disease activity. Several antibodies such as natalizumab, ocrelizumab, ofatumumab, and alemtuzumab are considered highly effective options for treating RRMS/RMS allowing for effective control of disease activity [10]. Ocrelizumab and to some extent also rituximab were shown to have a positive impact also on disease progression. Here, it seems that the subgroup of PMS patients with ongoing inflammatory activity benefit more than patients without disease activity [11]. Apart from efficacy, the risks of treatment with monoclonal antibodies deserve special attention [12, 13]. Especially during long-term therapy, the whole immune system will be affected by the specific intervention, regardless of the fact that monoclonal antibodies are focused on a specific molecular target. The long-term consequences of therapeutic modulation of the immune system are principally unpredictable, as illustrated by daclizumab, a monoclonal antibody directed against the interleukin-2-receptor. This antibody was withdrawn from the market and lost approval when serious adverse events became apparent [14–16].

## Anti-inflammatory Approaches for Treating PMS

### T Cells Versus B cells

From the early days of immunomodulatory therapy of MS with interferon-beta, it has been known that PMS is a more difficult therapeutic target than RRMS. Yet, even in those early days, there was evidence that PMS is not entirely resistant to immunomodulatory therapy if treatment is initiated sufficiently early [17, 18]. One of the most potent options for the treatment of active RRMS is natalizumab. Natalizumab acts by inhibiting alpha-4-integrin-mediated migration of lymphocytes, notably T-lymphocytes, from blood into the CNS. The therapeutic efficacy of alpha-4-integrin inhibition was first shown in a T-cell-mediated animal model, experimental autoimmune encephalomyelitis (EAE) [19]. It was only logical to test natalizumab also in patients with PMS. The ASCEND trial program investigated whether natalizumab was able to slow relapse-independent progression in patients with secondary progressive MS. In this placebo-controlled, randomized, double-blind study, 888 patients with SPMS participated. Of these, 439 were in the natalizumab cohort and 449 in the placebo group. The observation period of 2 years was followed by an open-label extension (OLE). Disappointingly, the ASCEND study was terminated after publication of blinded study results. Natalizumab did not reduce progression on the the primary multicomponent disability endpoint but only on its upper-limb component [20]. Specifically, there was no effect on walking deterioration as measured by the EDSS as well as the timed 25-foot walk (T25FW) as part of the multicomponent primary endpoint. However, for the third component of the primary endpoints, the 9-Hole Peg Test (9HPT), there was a significantly lower relative risk (minus 44%) of the natalizumab group developing upper extremity disability (15% vs. 23%; *p* = 0.001). This effect was independent of the detection of active inflammatory lesions on magnetic resonance imaging. Clinical relevance of this finding is possible, particularly in regard to upper extremity function preservation and independence [20]. Interestingly, these results are consistent with the IMPACT trial (interferon beta 1a in secondary progressive MS) [21]. Again, efficacy was seen only on the upper extremities with less relapse-independent progression but lack of efficacy on gait disturbance with shortening of walking distance [21]. Overall, a potential therapeutic effect of natalizumab purely on disability progression and independent of disease activity remains unclear. Based on the partly positive aspects, it can be assumed that the treatment duration may have been too short and that future studies should plan for a longer treatment duration [20] (Table 1).

**Table 1:** Monoclonal antibodies for progressive multiple sclerosis (table according to [6, 101, 103]

Monoclonal antibody	Study	Details	Primary endpoint	Key results	Reference
Phase III					
Natalizumab	Phase III SPMS (ASCEND)	MC, R, DB, PC, SD 2 years, 887 patients, MA(y) 47, MDoP(y) 5, eEDSS 3.0–6.5	Time to 3 month composite CDP	Negative (no treatment effect on multicomponent outcome, EDSS or the T25FW; but reduced 9HPT progression)	[20]
Rituximab	Phase II/III PPMS (OLYMPUS)	MC, R, DB, PC, 2 years, 439 patients, MA(y) 49, MDoP(y) 9, eEDSS 2.0–6.5	Time to CDP	Negative (no significant benefit in CDP for RIX vs. placebo)	[104]
Ocrelizumab	Phase III PPMS (ORATORIO)	MC, R, DB, PC, minimum 120 weeks, event driven study, 732 patients, MA(y) 45, MDoP(y) 6, eEDSS 3.0–6.5	Time to 12-week confirmed CDP	Positive (the relative risk of 12-week confirmed disability progression was significantly decreased by 24% (*p* = 0.03))	[29, 53]
	Phase III PPMS (O`Hand)	MC, R, DB, PC, 27 months, aim 1000 patients, eEDSS ≥ 3.0–8.0	Time to upper limb disability progression confirmed For at least 12 weeks	Recruiting	Analysis ongoing
	Phase III PPMS/SPMS (Consonance)	MC, open-label, single-arm, aim 900 patients, 4 years, eEDSS ≤ 6.5	Proportion of patients with no evidence of progression (NEP) on 6 month EDSS CDP	Recruiting	Analysis ongoing
Phase II					
Opicinumab	Phase II ON (RENEW)	MC, R, DB, PC, 32 weeks	Remyelination at 24 weeks, measured as recovery using full-field visual evoked potential (FF-VEP)	Negative (remyelination did not differ significantly between the opicinumab and placebo groups in the ITT population at week 24)	[105]
Temelimab	Phase II RMS (ProTEct-MS)	MC, R, DB, PC, 1 year, eEDSS 2.5–5.5	Mean overall response score (ORS): EDSS, T25FW, 9HPT-DH, 9HPT-NDH	Ongoing	Pre-study [106]
Elezanumab	Phase II PPMS/SPMS	MC, R, DB, PC, 123 patients, 1 year	Composite score of EDSS, T25FW, 9HPT	Completed	Analysis ongoing

*SC* single center, *MC* multicenter, *R* randomized, *DB* double blinded, *eEDSS* entry EDSS, *PC* placebo controlled, *SD* study duration, *MA(y)* mean age years, *MDoP(y)* mean duration of progression years

In addition to T cells, B cells have increasingly come into focus as important elements in the immunopathogenesis of MS, not least due to therapeutic developments. Thus, B cells were established as bidirectional interaction partners of T cells, both in the periphery and in the CNS [22–24]. This concept is supported by the high efficacy of B-cell depletion with anti-CD20 monoclonal antibodies [23–32]. CD20 is a surface molecule expressed by B cells at different stages of maturation. CD20 positivity is observed across different stages ranging from pre-B cells in bone marrow to short-lived plasmablasts. However, long-lived antibody-producing plasma cells are CD20 negative [32]. Notably, CD20 is not exclusive to B cells but is also present on a subset of T cells that could play a role in the pathogenesis of MS [33]. Several anti-CD20 therapeutic monoclonal antibodies were developed to achieve B-cell depletion. The antibodies differ in their structural features (chimeric, humanized, fully human antibodies), relative potency to elicit antibody-dependent cellular and complement-mediated cytotoxicity, and pharmacokinetics [34]. They also differ in the route of administration (intravenously or subcutaneously), infusion times, and the need for premedication [34].

What exactly is the the role of B cells and antibodies in MS pathogenesis? Obviously B cells are the source of antibodies, including CSF-specific oligoclonal bands and immunoglobulins deposited in MS lesions [35]. It is not clear, however, if intrathecally produced antibodies which include antibodies directed against cellular debris are pathogenic [36]. It is therefore likely that the therapeutic efficacy of CD20-mediated B cell depletion is brought about by inhibition of B-cell functions other than antibody production, including antigen presentation to T cells, and production of various cytokines and chemokines [37]. The importance of these intercellular interactions appears to be particularly high in active early stages of MS. In later or progressive stages, plasma cell infiltrates, which are insensitive to anti-CD20-mediated depletion, might play an increasingly important role [38]. Nevertheless, clinical efficacy of CD20 antibody therapy was shown in both relapsing and progressive phases of MS. It should be noted that anti-CD20 antibodies differ in their molecular and pharmacological properties. For example, rituximab acts mostly by complement-dependent B-cell depletion, whereas ocrelizumab acts mostly via antibody-dependent cellular cytotoxicity [39]. These distinctive properties not only impact the mechanisms and efficacy of monoclonal CD20 antibodies, but also the required dosing [40]. Although current MS dosing regimens result in near complete depletion of circulating B cells, dose-dependent differential kinetics of B cell reconstitution are evident. One possible interpretation could therefore be that near-complete peripheral B-cell depletion may be accompanied by varying degrees of depletion in tissues, immune cell niches, or secluded compartments such as the CNS [41].

However, why and to what extent the described differential mechanisms of monoclonal CD20 antibodies, which are administered outside the CNS, may contribute to their beneficial effects on MS progression remains unresolved. It is also not clear whether the efficacy of anti-CD20-mediated depletion in the CNS correlates with the therapeutic effect on disease progression. In progressive MS, there is an increasing “compartmentalization” of inflammation within the CNS, with accumulation of clonally expanded B cells in meningeal B-cell follicle-like structures. They provide a niche in the CNS which supports and maintains pathogenic B-cell function [42, 43] and could be instrumental in driving chronic progression [42]. B-cell-associated pathogenetic features include cortical lesions as well as diffuse microglial activation [43].

Depletion of peripheral B cells is accompanied by a marked decrease in B cells not only in the cerebrospinal fluid (CSF) but also in the perivascular spaces of the brain [44]. Relevant crossing of CD20 antibodies across the intact blood–brain barrier is unlikely [45]. Therefore, it seems that monoclonal CD20 antibodies can only be effective if systemic depletion of peripheral B cells occurs during a phase of the disease course when peripheral immune cells are actively recruited to the CNS [46]. Thus, an obvious therapeutic approach could be the intrathecal administration of monoclonal anti-CD20 antibodies. The efficacy of such therapeutic strategies is currently investigated with ongoing animal research and clinical studies [46].

### Rituximab

Rituximab is a chimeric monoclonal CD20 antibody that is used off-label in many places for the treatment of MS. This antibody has not been approved by the FDA (US Food and Drug Administration) or European Medicines Agency (EMA) for the treatment of MS [47–51]. However, the efficacy of rituximab has been studied in several trials in MS. In the 96-week phase II/III OLYMPUS study, a randomized controlled trial in primary progressive multiple sclerosis (PPMS), rituximab did not significantly improve confirmed disability progression (CDP, *p* = 0.14) or reduce brain atrophy rate (*p* = 0.62) (Table 1). However, there was significantly less T2 hyperintense lesion volume increase at week 96 (*p* < 0.001) compared with placebo [28].

Subgroup analyses showed that rituximab could delay time to CDP in younger PPMS patients (age < 51 years) or in patients with Gd-enhancing lesions at baseline. Thus, a positive prediction regarding future treatment response could be derived from subgroup analyses [52] and from very consistent ORATORIO experiences. The ORATORIO study evaluated the safety and efficacy of ocrelizumab in PPMS. ORATORIO is an international, multicenter, double-blind, randomized, placebo-controlled phase III study [29, 53].

To achieve depletion of CNS-resident B cells, intrathecal administration of rituximab has been explored as a mode of application. In a trial of intrathecal rituximab in PMS patients with MRI evidence of leptomeningeal contrast enhancement, there was a profound reduction of peripheral B cells and transient reduction of B cells in the CSF. However, the number of contrast-enhancing leptomeningeal sites did not change following treatment [54].

### Ocrelizumab

The humanized anti-CD20 monoclonal antibody ocrelizumab was approved at a dose of 600 mg i.v. twice yearly for the treatment of PPMS with evidence of disease activity in March 2017 (FDA) and January 2018 (EMA). The antibody targets CD20-expressing lymphocytes, mostly B cells but also a smaller subset of T cells [32, 33].

The EMA has licensed ocrelizumab based on the pivotal data for the treatment of adult patients with early PPMS, characterized by disease duration and degree of disability, as well as imaging features typical of inflammatory activity [55, 56]. The approval was based on the ORATORIO trial and on the study population investigated (Table 1). ORATORIO enrolled 732 PPMS patients and treated them with either ocrelizumab or placebo every 6 months for at least 120 weeks [26]. The study population represented early-stage PPMS patients, i.e., 18 to 55 years of age (inclusive), with an EDSS of 3.0 to 6.5 at the time of screening, and a duration of disease since the onset of first MS symptoms of less than 10 years (for patients with an EDSS of ≤ 5.0 at screening) or less than 15 years (for patients with an EDSS of > 5.0 at screening). The implication for clinical practice is that evidence of inflammatory activity, defined by Gd-uptaking T1 lesions and/or active [new or enlarging] T2 lesions), should be obtained by MRI in all patients who are considered candidates for treatment with ocrelizumab. Patients older than 55 years were not studied in the clinical trials [57]. In the pivotal study, ocrelizumab met both the primary endpoint (reduction in the risk of disability progression confirmed at 12 weeks) and the secondary endpoints.

The proportion of patients with CDP in the EDSS score at 12 weeks was reduced by 24% compared with placebo (significant reduction in 12-week CDP (*p* = 0.03) and its confirmation in 24-week CDP (*p* = 0.04)) [26]. CDP at 24 weeks was defined as follows:

(1) Increase in EDSS score, (2) ≥ 20% increase in time to complete the 9-Hole Peg Test [9HPT], (3) ≥ 20% increase in time to complete the timed 25-foot walk [T25FW], and composite progression, defined as the first confirmed occurrence of any of these three (1–3) individual measures. In addition, time to need for a wheelchair (EDSS ≥ 7) was considered [29].

Subanalyses of hand function (9HPT) and walking ability (T25FW) confirmed the superiority of ocrelizumab with significantly less worsening in these motor function subscores [26]. Ocrelizumab significantly reduced the risk of disability progression, less T2 lesion volume for ocrelizumab-treated patients (minus 92% vs. placebo; *p* < 0.001), and brain atrophy compared with placebo (*p* = 0.02). However, this efficacy was driven by the subgroup of younger study participants (< 40 years) with disease activity, e.g., disease activity on MRI (≥ 1 Gd-enhancing lesions) [26, 58]. As mentioned before this is consistent with experiences from the OLYMPUS trial (rituximab in PPMS) [28].

After the double-blind phase of the pivotal study, study participants could enter the optional open-label extension phase (OLE). Patients previously treated with ocrelizumab remained on treatment (initial ocrelizumab cohort), and patients from the placebo group were switched to the ocrelizumab treatment arm (initial placebo cohort).

Five hundred twenty-seven patients in the ORATORIO study program (97%) entered the OLE phase, and of these patients, 86% were analyzed. At 6.5 years, the proportion of patients with disability progression was lower in the initial OCR cohort vs. initial placebo cohort. EDSS progression was seen in 13.1% fewer patients in the initial OCR cohort (*p* = 0.0018), 12.5% fewer had relevant worsening in the 9HPT (*p* = 0.0035), and 7.5% fewer had relevant worsening in the T25FW (*p* = 0.058). Compound progression was seen by 10.1% fewer in the initial OCR cohort (*p* = 0.0023). Paraclinically, T2 lesion volume (0.45% vs. 13%, *p* < 0.0001) and T1 hypointense lesion volume (36.68% vs. 60.93%, *p* < 0.0001) were significantly reduced. Over the entire period, the serious adverse event rate was 12.6 per 100 patient-years; the most common serious adverse event was infection at 4 per 100 patient-years. No new safety signals occurred over 6.5 years compared with the double-blind phase of ORATORIO [29].

## Future Prospects of B-Cell Targeting in PMS

### Inebilizumab

Direct depletion of CD19-positive cells may represent another B-cell targeting therapeutic strategy. CD19 is a member of the Ig superfamily and is involved in signal transduction following B cell receptor activation, in the regulation of B cell activation and humoral immunity [59]. As a therapeutic approach, CD19 is of interest because it is expressed on a greater range of B-cell lineage members, including pro-B cells and plasmablasts [59], than is CD20.

Inebilizumab is a glycosylated, afucosylated anti-CD19 antibody. The results of a 24-week phase I randomized controlled trial in patients with RRMS compared with placebo are available [60]. An investigation in PMS is currently pending.

## Neuroprotective and Remyelination-Fostering Strategies

### Therapeutic Targeting of Microglia and Oligodendrocytes

Apart from oligodendrocytes, microglia has emerged as a prime potential target for treating PMS. Histopathologically, PMS is associated with inactive, but also with chronically active, “smoldering” lesions, which are surrounded by a rim of microglial activation [61]. Moreover, diffuse microglial activation can occur independent of focal lesions [62, 63]. Positron emission tomography (PET) could represent one possibility of monitoring microglial activation in the normal appearing white matter (NAWM). Here, the upregulation of the mitochondrial translocator protein TSPO provides a marker that can be visualized with modern tracers [64–66].

The exact pathophysiological function of microglia in progression remains speculative. Expression of proinflammatory cytokines [67] and also the contribution of microglia to mitochondrial damage may be be relevant [68]. It should be noted, however, that microglia may also contribute to repair and remyelination via mechanisms of phagocytosis and production of anti-inflammatory cytokines [69, 70] promoting the recruitment of oligodendrocyte precursor cells (OPC) to the lesion site [71, 72].

In view of these complexities, a differentiated treatment strategy is required for promoting neuroprotection and remyelination. The overall goal is to promote remyelination capacity, as well as to reduce proinflammatory microglial activity [73]. Presently, however, no singular specific target is known that fulfills both requirements. Another consideration, which is relevant for antibody-based therapies, is that neuroprotective and remyelination-fostering therapies would have to reach the CNS in sufficiently high concentrations, which may be more easily achieved with small molecule drugs.

### Opicinumab

The transmembrane cell surface glycoprotein LINGO-1 has a significant role in controlling oligodendrocyte precursor proteins (OPCs) and neurons [74–77]. LINGO-1 is known to be upregulated in MS lesions. It has been shown in vitro and in animal studies that blockade of LINGO-1 can lead to increased axonal myelination as well as improvement in clinical scores [78, 79]. This was the basis for investigating the monoclonal antibody opicinumab, a fully humanized anti-LINGO1-antibody, in several clinical trials [80–85].

In the RENEW trial, a randomized, placebo-controlled, multicenter phase II study (33 treated, 36 placebo) in patients with a first unilateral acute optic neuritis episode, no benefits regarding remyelination were shown in an intention-to-treat analysis [86] (Table 1). Visual outcomes, specifically visual evoked potentials (VEP), optical coherence tomography (OCT), and MRI, were examined [83]. However, a post hoc analysis indicated that older patients in particular may benefit from therapy with opicinumab [82].

The SYNERGY trial also failed to show a significant beneficial treatment advantage of the opicinumab group over the comparison cohort. This was a randomized, double-blind, placebo-controlled, dose-ranging phase II study (*N* = 419) to investigate the clinical efficacy, safety, and pharmacokinetics of opicinumab in which patients were treated with intramuscular interferon beta-1a in combination with either placebo or a variable dose of opicinumab (3/10/30/100 mg/kg) [81]. The primary endpoint was the percentage of participants with confirmed clinical improvement over 72 weeks of treatment. The study was negative overall, although trends in some subscores were apparent [81]. There was no dose-linear improvement in disability for the opicinumab treatment arm. However, compared to the RENEW trial, RRMS patients with younger age, shorter disease duration, and less brain atrophy showed some benefit. Overall, the remyelination potential of opicinumab and the reasons for the negative results of the study remain unclear at this time. Based on the study design, the study could have been underpowered, the selection of patients might not have been optimal, and poor penetration of antibodies through the blood–brain barrier could have contributed to the results. Furthermore, the expression of LINGO-1 may be variable, depending on disease activity [87].

## Outlook (I): Tackle the Immune Cell Niche

In addition to the therapeutic development of additional and well-established antibodies, with so-called small molecules, a new option of treating PwPMS is emerging. One possible therapeutic approach is the inhibition of Bruton’s tyrosine kinase (BTK) [88]. BTK regulates the activation, proliferation, and differentiation of B cells into plasma cells [89, 90]. BTK is a cytoplasmic kinase expressed on some cells of the hematopoietic cell lineage including B cells and myeloid cells. BTK is not expressed on T or NK cells [91]. As “small molecules,” BTK have the advantage that they can cross the blood–brain barrier more easily than antibodies, thereby allowing for more effective targeting of the CNS-resident (compartmentalized) B-cell population [89, 90].

Different BTK inhibitors are currently being explored in clinical trials (see [89] for a comprehensive review). Ideally, a BTK inhibitor not only should easily cross the BBB, but it also should bind BTK in a highly selective but reversible manner. One example is evobrutinib. Evobrutinib was able to meet the primary endpoint (number of T1 gadolinium-enhancing lesions) in a 24-week phase II clinical trial comparing oral evobrutinib at various doses with placebo or dimethyl fumarate in patients with RRMS or active SPMS, but showed no efficacy on disability progression [92]. As presented by Gheen et al. [93], fenebrutinib is currently being studied in a phase III trial in PPMS (examination of fenebrutinib, a highly selective BTKi, on disease progression of multiple sclerosis). Another BTK inhibitor, tolebrutinib, was studied at different doses in a 16-week phase IIb study in RRMS. Also here, efficacy on disease activity was noted. However, based on this study, no conclusions can be drawn regarding potential efficacy in PMS. A study in PPMS (PERSEUS) and non-active SPMS (HERCULES) is currently recruiting (ClinicalTrials.gov Identifier: NCT04458051; ClinicalTrials.gov Identifier: NCT04411641). Whether BTK inhibitors have neuroprotective in addition to their anti-inflammatory properties remains to be elucidated [94].

## Outlook (II): New Approaches Regarding Study Designs, Strategies, and Objectives

It remains to be evaluated whether the traditional outcome measures such as disability progression measured with the EDSS remain the most suitable tool for studies in PMS. It is expected that future studies will need to focus on more specific biological or paraclinical outcome measures [95]. These could include novel cell-specific markers of inflammatory and degenerative mechanisms as well as a broader selection of already existing paraclinical markers. Besides electrophysiological investigations such as somatosensory (SEP), motor (MEP) and visual evoked potentials (VEP), modern magnetic resonance imaging (MRI) parameters, and specific positron emission tomography (PET), examinations could play a role in future study designs [95]. Last but not least, optical coherence tomography (OCT) could be potentially suitable as a marker of retinal neuroaxonal degeneration [96]. In this regard, the International Progressive MS Alliance (https://www.progressivemsalliance.org/) has recently published a proposal of a core data set that could be used as a standard for future trial designs in PwPMS [95]. This includes the established clinical scores (EDSS, upper limb dexterity), paraclinical measures (Neurofilament light [NfL] protein, brain atrophy, VEP and/or OCT), immune treatment response markers (sCD21, SCD27, sCD14, CXCL13, BAFF), and non-hypothesis driven measures like peripheral transcriptomics [95].

Other important aspects include the selection of the appropriate study population. For example, PwPMS should be selected taking into account disease dynamics during the disease course, but also the sex distribution within the study population, which should correspond to the sex distribution in the targeted MS population [95]. The study design is also critical for the success of a therapeutic development. It is questionable whether the complex interaction between inflammatory and neurodegenerative processes can be adequately addressed with a monotherapy. Of course, cumulative risks of polytherapy, tolerability, and costs to healthcare systems must be kept in mind for any development. However, combination therapy including immunosuppressants, but also remyelination or neuroprotective treatment strategies should be considered in the future. Regarding the design of PMS treatment trials, modern study designs make use of adaptive, enrichment, futility, or crossover design elements [95].

Even with improved study designs, the heterogeneity of MS populations continues to contribute an unavoidable element of unpredictability. Viewed in terms of pathophysiology, therapeutic targets should include proinflammatory microglia, active astrocytes, mitochondrial dysfunction, and oxidative stress. At the same time, the ability to remyelinate should be supported [97]. Related to the remyelination capacity of PwMS, it is known that there are two groups of patients, those considered good and those classified as poor/insufficient remyelinators [98]. Influencing factors could be age, duration of disease, presence of oligodendrocyte progenitor cells (OPCs), genetic factors, and environmental factors [99]. Interestingly, gender also appears to play a role: although more women than men are affected by MS overall, the disease course in men appears overall more aggressive [100].

In conclusion, it seems promising to investigate strategies to promote myelin repair from endogenous as well as exogenous cell sources. In addition, the different factors influencing remyelination should be therapeutically addressed to support the spontaneous repair of demyelinated axons [97].

## Conclusions

The currently existing therapeutic options for the treatment of PMS remain inadequate. However, recent advances in understanding the pathophysiology of PMS offer hope for new innovative therapeutic options. Effective therapy must address the compartmentalized inflammation in the CNS. Furthermore, the classical requirement for immunotherapy in RRMS, namely early initiation of an effective therapy, is equally relevant for treating PMS. However, the data available so far on neuroprotection and remyelination in PMS do not yet allow definition of unequivocal target priorities. What seems clear, however, is that the principle of early therapy applies not only to RRMS but also to PMS. Neuroprotection is most effective when there are still myelinated axons to protect, and remyelination is most effective, when there are still demyelinated axons to remyelinate [101].

In particular, PMS is considered a very demanding challenge in the care of MS patients [101, 102]. Future therapy strategies should therefore always be multimodal. Besides possible antibody therapy options for anti-inflammation, neuroprotection, or remyelination, symptomatic therapy should also play a crucial role. Measuring the success of therapy by practical criteria is of key importance. One example is NEPAD, an acronym standing for “no evidence of progression or active disease,” i.e., “Lublin not active and without progression” [29, 57]. Last but not least, special attention must always be paid to benefit/risk analysis. Safety of treatment and strategies for dealing with adverse effects are at least as important as efficacy.

## Supplementary Information

Below is the link to the electronic supplementary material.

Supplementary file1 (PDF 1225 KB)
